# A benchmarking framework for the accurate and cost-effective detection of clinically-relevant structural variants for cancer target identification and diagnosis

**DOI:** 10.1186/s12967-024-04865-w

**Published:** 2024-01-16

**Authors:** Guiwu Zhuang, Xiaotao Zhang, Wenjing Du, Libin Xu, Jiyong Ma, Haitao Luo, Hongzhen Tang, Wei Wang, Peng Wang, Miao Li, Xu Yang, Dongfang Wu, Shencun Fang

**Affiliations:** 1 Department of Gastrointestinal Surgery, The Eighth Affiliated Hospital, Sun Yat-Sen University, Shenzhen, China; 2 Department of Radiotherapy, Qingdao Central Hospital, University of Health and Rehabilitation Sciences, Qingdao, China; 3 Department of Radiotherapy, Shanxi Province Cancer Hospital/Shanxi Hospital Affiliated to Cancer Hospital, Chinese Academy of Medical Sciences/Cancer Hospital Affiliated to Shanxi Medical University, Taiyuan, China; 4 Department of Orthopedic Surgery, National Cancer Center/National Clinical Research Center for Cancer/Cancer Hospital, Chinese Academy of Medical Sciences and Peking Union Medical College, Beijing, China; 5 Department of Respiration, Nanjing First Hospital, Nanjing Medical University, Nanjing, China; 6 Shenzhen Engineering Center for Translational Medicine of Precision Cancer Immunodiagnosis and Therapy, YuceBio Technology Co., Ltd., Shenzhen, China; 7 Department of Respiratory Medicine, Nanjing Chest Hospital, The Affiliated Brain Hospital of Nanjing Medical University, Nanjing, China

**Keywords:** Structural variant, Computational framework, Performance assessment, Decision model, Clinical application, Cancer target

## Abstract

**Background:**

Accurate clinical structural variant (SV) calling is essential for cancer target identification and diagnosis but has been historically challenging due to the lack of ground truth for clinical specimens. Meanwhile, reduced clinical-testing cost is the key to the widespread clinical utility.

**Methods:**

We analyzed massive data from tumor samples of 476 patients and developed a computational framework for accurate and cost-effective detection of clinically-relevant SVs. In addition, standard materials and classical experiments including immunohistochemistry and/or fluorescence in situ hybridization were used to validate the developed computational framework.

**Results:**

We systematically evaluated the common algorithms for SV detection and established an expert-reviewed SV call set of 1,303 tumor-specific SVs with high-evidence levels. Moreover, we developed a random-forest-based decision model to improve the true positive of SVs. To independently validate the tailored ‘two-step’ strategy, we utilized standard materials and classical experiments. The accuracy of the model was over 90% (92–99.78%) for all types of data.

**Conclusion:**

Our study provides a valuable resource and an actionable guide to improve cancer-specific SV detection accuracy and clinical applicability.

**Supplementary Information:**

The online version contains supplementary material available at 10.1186/s12967-024-04865-w.

## Background

Structural variations (SVs) are most generally defined as large-scale genomic changes encompassing at least 50 base pairs, which include insertions, inversions, deletions, duplications/amplifications, and translocations of genomic segments [1]. Previous studies have shown that SVs play critical roles in tumor development, progression, and resistance to treatment by changing gene copy numbers, activating oncogenes, disrupting suppressor genes, or creating novel gene fusions [1–3]. In addition, at least 30% of cancers have a known pathogenic SV that can be used for clinical diagnosis or therapy [4–9]. For example, the first-generation tropomyosin receptor kinase inhibitors Entrectinib and Larotrectinib, have been approved by the Food and Drug Administration (FDA) in the United States to target NTRK fusions in adult and pediatric patients with solid tumors [10]. The mechanism of both drugs involves selectively inhibiting the abnormal signaling pathways driven by NTRK gene fusions, leading to tumor regression and improved patient outcomes (response rates > 75%) [10]. Hence, accurate detection of tumor-specific SVs with high- and/or multi-evidence levels in cancer patients is necessary.

The advancement of next-generation sequencing (NGS) technologies has enabled the large-scale discovery of SVs, and uncovering their relationship to cancer. Despite the significant advantages of whole genome/whole exome sequencing (WGS/WES) in detecting SV on a genome-wide scale, their utility in clinical testing has been questioned mainly due to the low-cost effectiveness and the uncertain consequences of the large number of variants with unknown significance. In clinical practice, only actionable mutations that are clinically-relevant and would benefit a specific therapy are worth detecting. In such cases, applying targeted panels is the preferred option for clinical SV detection, offering higher coverage of target sites and lower cost and time.

Accurately SV calling based on the target panel is a critical step upon which almost all downstream analysis and annotation processes rely. Numerous studies employ commonly used algorithms in detecting SVs, such as Delly [11], Lumpy [12], GRIDSS [13], SvABA [14], and Manta [15]. These algorithms provide relatively high accuracy calls for SVs but their efficacy varies across the sizes and types of SVs [16]. In addition, rare research focused on improving the detection accuracy of clinically-relevant SVs in targeted NGS panels, mainly due to the lack of ground truth for clinical specimens.

In this study, based on tumor samples of 476 patients combined with standard materials, we evaluated the strengths and weaknesses of SV detection algorithms and developed a computational framework for accurate and cost-effective detection of clinically-relevant SVs. The key question our work mainly answers is how to best integrate cancer SV targets identified by panel sequencing into the clinical diagnostic and treatment pathways.

## Methods

### Sample collection

The data of 476 cancer patients used in this study was collected in different periods, which were divided into three main cohorts according to the time of collection and the type of detection. From July to November 2021, a total of 329 patient samples were first collected. Among these samples, 5 samples without any target SVs identified by common SV callers and confirmed by the Integrative Genomics Viewer (IGV) tool [17] were used to generate the simulation data and evaluate the performance of common SV callers. The remaining 324 patients’ data was used as cohort 1 to construct and validate the random-forest decision model. For cohort 2, a total of 60 patients were further enrolled in December 2021, which was used to validate the decision model as an independent cohort. For cohort 3, 87 patients who underwent both panel sequencing and IHC and/or FISH experiments between January to March 2022 were enrolled to further validate the performance of the benchmarking framework at the experiment level.

### Targeted next-generation sequencing (NGS) and data processing

Targeted next-generation sequencing for Formalin-Fixed and Paraffin-Embedded (FFPE) tumor tissue and matched peripheral blood samples was carried out on 476 cancer patients across various cancer types, mainly including lung, intestine, stomach, liver, breast, ovary, and skin cancers. Genomic DNA was extracted from tumor and blood samples using the GeneRead DNA FFPE and DNA blood mini kit (QIAGEN, GER), respectively. Targeted NGS libraries were prepared using the custom panel (Yucebio, China), which included high evidence levels of clinically-relevant SVs from 27 genes. Then, the libraries were sequenced on the MGISEQ platform with 100 bp paired-end reads following standard procedures. The medium depth of coverage was 1773× for tumors and 878× for matched blood controls.

SOAPnuke (v1.5.6) was applied to remove sequencing reads with adapters, low-quality, and/or > 10% N rate. The remaining high-quality reads were aligned to the human reference genome (version hg19/GRCh37) using the Burrows-Wheeler Aligner program (BWA, version 0.7.12). Common SV callers were applied to identify SVs, including Delly (v0.8.7), SvABA (v1.1.0), Manta (v1.6.0), and Lumpy (v0.2.13).

### Performance assessment of SV callers based on simulation data

To assess the performance of SV callers, we performed variant simulation on five patients’ real-sequencing data (which did not contain any target SVs identified by common SV callers and confirmed by the IGV tool) by VarBen (v1.0). Custom SVs (Additional file 1: Table S1) were utilized in the generation of variant simulation data. In addition, we also generated five variant allele frequency (VAF) gradients (0.5%, 1%, 2%, 5%, and 10%) for each data. The remaining parameters of VarBen were specified as “–r ucsc.hg19.fasta –aligner bwa –alignerIndex ucsc.hg19.fasta –seqer BGI –mindepth 100 –minmutreads 3 –readlength 100 –p 20”. The average detection rate was used to evaluate the performance of SV callers and the student’s t-test was performed to compare the difference between the two groups.

### Random-forest decision model for prediction of bona fide SVs

To construct the random-forest decision model for the prediction of bona fide SVs, we first need to label SVs detected by the best-performance SV caller obtained in the previous step as either true or false positives. The IGV tool [17], which can provide high-performance data visualization and exploration on standard desktop systems, was applied to visually inspect each variant and determine whether it was a true or false positive. Expert review was processed according to the characteristics of soft-clip/split reads, reads number, signal strength, and other features of the breakpoints. At least two independent bioinformatic engineers and interpretation experts examined the results, marking any inconsistent SV under analysis as a false positive.

Based on the expert-reviewed true- or false-positive information of detected SVs, the random-forest algorithm was performed with ten-fold cross-validation to construct a random-forest decision model to improve the accuracy of detected SVs. Initially, we performed a preliminary parameter optimization by invoking the hyperparameter random search method over a larger range of hyperparameters in the training set. The hyperparameters involved in the model optimization process, include n_estimators, max_features, max_depth, min_samples_split, min_samples_leaf, and bootstrap. When conducting the hyperparameter randomization search, the range of settings for each hyperparameter is as follows: n_estimators (50, 100, 150, …, and 3000, with 60 evenly spaced values); max_features (‘auto’ and ‘sqrt’); max_depth (‘None’, 10, 20, 30, …, 500, with 50 evenly spaced values); min_samples_split (2, 5, and 10); min_samples_leaf (1, 2, 4, and 8); bootstrap (True and False). Then, the best hyperparameters of the random search were acquired, including n_estimators = 200, max_features = sqrt, max_depth = 40, min_samples_split = 5, min_samples_leaf = 1, and bootstrap = True. Subsequently, a hyperparameter grid search was conducted, and the ultimate model was developed. The range of settings for each hyperparameter is as follows: n_estimators (180, 181, 182, …, 220, with 41 evenly spaced values); features = sqrt; max_depth (30, 31, 32, …, 50, with 21 evenly spaced values); min_samples_split (3, 4, 5, 6, and 7); min_samples_leaf = 1; bootstrap = True. Finally, the best hyperparameters of the grid search were obtained, n_estimators = 186, max_features = sqrt, max_depth = 40, min_samples_split = 6, min_samples_leaf = 1, and bootstrap = True.

Two cohorts involving 384 patients were utilized to construct and validate the random-forest decision model. In Cohort 1 (n = 324), all detected SVs were randomly divided into a training set and a testing set 1 at a 4:1 ratio. The training set was applied to construct the random-forest decision model. Testing set 1 and testing set 2 (from Cohort 2, n = 60) were utilized as internal and external validation sets to assess the reliability of the model, respectively. To evaluate and validate the performance of the model, four metrics, based on the confusion matrix, including accuracy (Eq. 1), precision (Eq. 2), recall (Eq. 3), and F1 score (Eq. 4), were employed in both the training and two testing sets.

1 $$Accuracy = \frac{TP + TN}{{TP + FP + TN + FN}}$$

2 $$Precision = \frac{TP}{{TP + FP}}$$

3 $$Recall = \frac{TP}{{TP + FN}}$$

4 $${\text{F}}1{\text{ score}} = 2 \times \frac{Recall \times Precision}{{Recall + Precision}}$$

### Confirmation by standard materials

To demonstrate the robustness of the model, we procured standard materials (Genewell Bio, China) with custom SVs (Additional file 1: Table S2). Two VAF gradients (1% and 2%) and five biological replicates for each VAF gradient were generated. These standards were generated by human cancer cell lines. In addition, we also procured mutation-free samples (Genewell Bio, China), which served as benchmark data when detecting SVs of standard materials. The recall metric was applied to assess the performance.

### Immunohistochemistry

Tissue blocks were cut into 4-µm-thick slides and subjected to antigen retrieval using an Automatic Antigen Repair Instrument (LBP-5196-II, LBP Medicine Science & Technology, China) for 30 min and 98 °C with EDTA buffer. Then, the slides were stained using an Automatic Immunocytochemical Staining Machine (LBP, LBP Medicine Science & Technology, China). All slides were examined by two independent qualified pathologists.

### Fluorescence in situ hybridization

The FISH test was performed on 4-μm-thick FFPE tissue slides with RET (LBP Medicine Science & Technology, China), ROS1 (LBP Medicine Science & Technology, China), and ALK (HealthCare, China) break-apart FISH Probes Kit according to the manufacturer's instructions on StatSpin ThermoBrite Elite (S500-24, Abbott, USA). The fluorescence signals were examined with a fluorescence microscope (BX53, Olympus, Japan). At least 100 tumor cells per specimen were scored by 2 professional pathologists. The gene-fusion-positive cells were defined as those with the separation of the green and red signals or the presence of an isolated 3ʹ signal (3ʹ probe of ALK is red, 3ʹ probes of RET and ROS1 are green). The sample was considered positive if the proportion of positive cells was greater than 15%.

## Results

### Overview of the framework

**Fig. 1:**
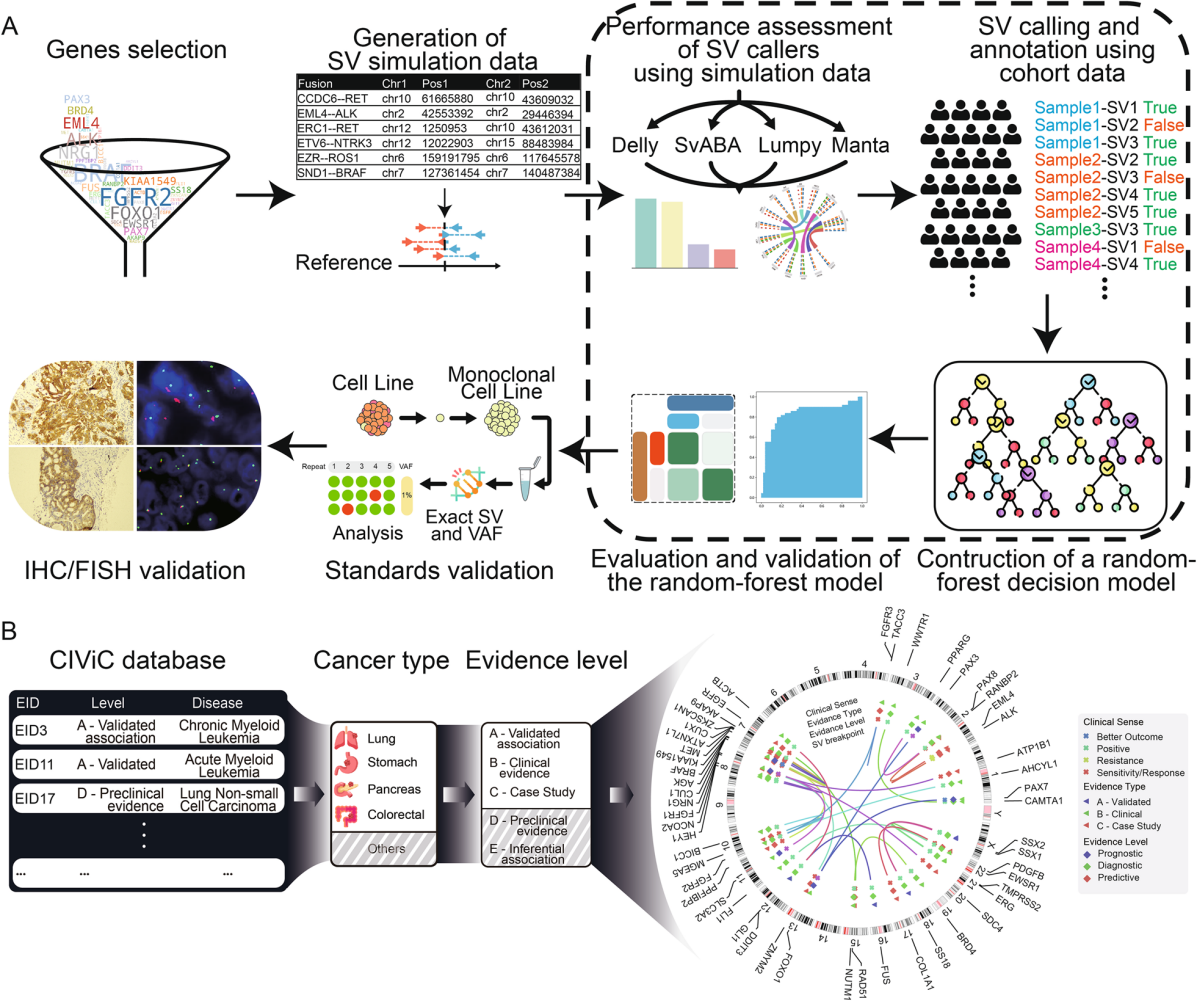
Overview of study. **A** Workflow of the study. **B** Workflow of obtaining 27 genes from the CIViC database

The current framework is mainly divided into five parts (as shown in Fig. 1A): (1) Screening and selection clinically-relevant SVs with high evidence levels; (2) Insert SV events into raw sequencing data of cancer patient samples and generate simulated data; (3) Evaluate the performance of common SV algorithms and identify the optimal method for analyzing custom panel sequencing data; (4) Construct the random-forest decision model to improve the accuracy of SV detection; (5) Verify the performance of the framework based on standard materials and classical experiments. Based on the cancer types and evidence levels of SVs provided by the Clinical Interpretation of Variants in Cancer (CIViC) database, 27 genes were selected with evidence of C-level or higher, meaning that whose SVs had been reported to be associated with cancer treatment or had undergone or completed clinical trials of targeted drugs (Additional file 1: Table S3, Fig. 1B). A total of 61 clinically-relevant SVs on these genes were used for further analysis, including 4 prognostic, 20 diagnostic and 37 predictive SVs according to their evidence levels (Additional file 1: Table S3).

### Performance assessment of SV detection methods

**Fig. 2:**
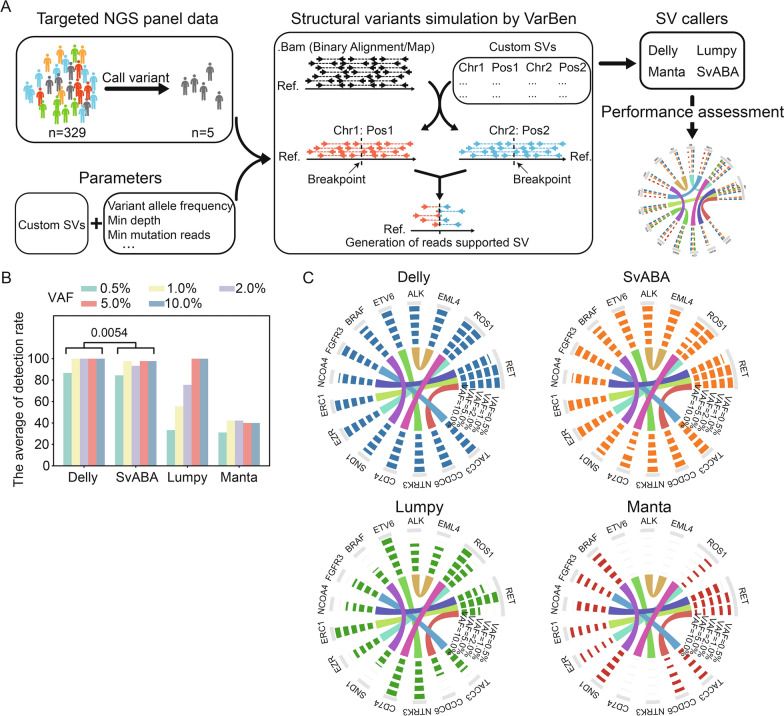
Performance assessment of SV callers. **A** The generation of SV simulation data. **B** The average detection rate of SV callers. **C** Circos plots exhibit the detection rates of each custom SV in SV simulation data for Delly (blue), SvABA (orange), Lumpy (green), and Manta (red). Each connection in the center of the circos plot represents the fusion of genes at both ends. The concentric rings proceed from outermost to innermost, signifying VAF values of 0.5, 1.0, 2.0, 5.0, and 10.0, respectively. The height of the barplot on each ring denotes the detection count of the corresponding SV

Based on raw targeted sequencing data from 329 cancer patients enrolled from July to November 2021, common SV callers (Delly, SvABA, Lumpy, and Manta) were utilized to detect clinically-relevant SV events. The results showed that five patients did not detect the target SVs, which was further confirmed by manual examination based on the IGV tool. Then, a read editing-based variant simulator VarBen was adopted to generate the SV simulation data by incorporating the custom-defined SV events into the raw sequencing data of these patients (Additional file 1: Table S1, Fig. 2A). In addition, five VAF gradients (0.5%, 1%, 2%, 5%, and 10%) for each simulation data were generated.

According to SV detection results, we found that when the mutation frequency gradients were equal to 5.0% and 10.0%, Delly and Lumpy achieved a higher average detection rate (equal to 100%) than the other two software (Fig. 2B). However, the average detection rate of Lumpy dramatically decreased as VAF gradients declined from 2.0% to 0.5%. Delly remained the top performer when VAF gradients were equal to 0.5% (86.67%), 1.0% (100%), and 2.0% (100%). Furthermore, Delly maintained an average detection rate > 86% when the VAF gradients dropped to 0.5%. SvABA also achieved a relatively high average detection rate of 84.44% in the 0.5% VAF gradients but significantly lower than Delly (*P* = 0.0054). In comparison, the other two SV callers exhibited an average detection rate of less than 35%. In addition, we compared the performance of the SV callers in each SV event and found that Delly and SvABA successfully detected most parts of gene fusion events in all VAF gradients (Fig. 2C). Especially, Delly exhibited a higher average detection rate in CD74-ROS1 fusions and NTPK3-ETV6 fusions than SvABA. Taken together, Delly is utilized in the first step of our framework for detecting SVs in targeted NGS panel data.

### Random-forest decision model can effectively improve the true positives

**Fig. 3:**
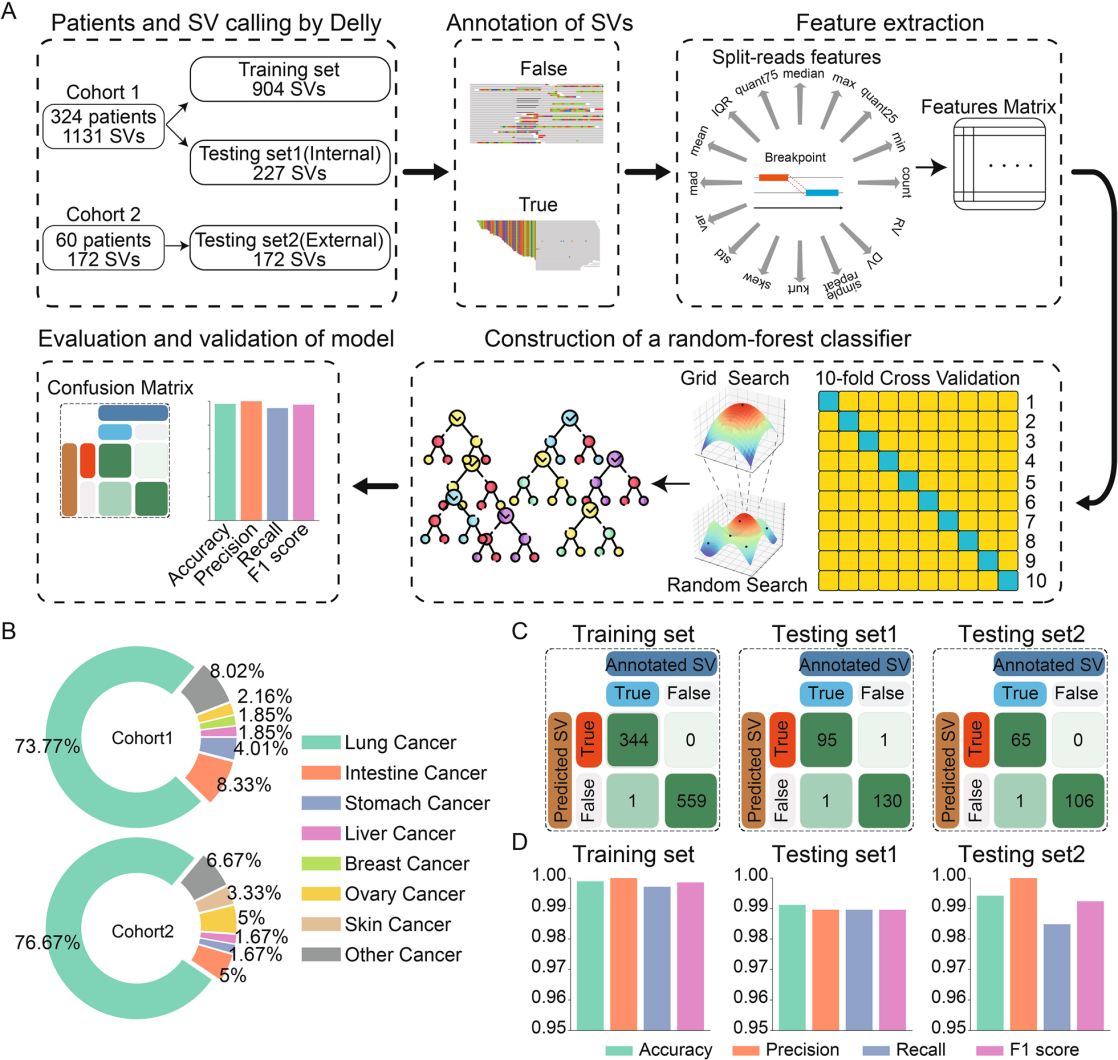
Construction of the random-forest decision model for prediction of bona fide SVs. **A** Workflow of the random-forest decision model construction. **B** The distribution of cancer types in Cohort 1 (Up) and Cohort 2 (Down). **C** Confusion matrixes in the training set (left), testing set1 (middle), and testing set2 (right). **D** Performance of random-forest decision model in the training set (left), testing set1 (middle), and testing set2 (right)

Further manual verification of the SV detection results reported by Delly revealed a considerable percentage of false positives, which prevented the further application of these results to clinical practice. To improve the true positive of SVs, we constructed a random-forest decision model based on 1131 SV events identified from 324 cancer patient samples (Cohort 1, Additional file 1: Table S4), which were further randomly divided into a training set (904 SVs) and a testing set (227 SVs) at a 4:1 ratio (Fig. 3A). Several cancer types were involved in the patient cohort, with relatively high percentages of lung, intestine, stomach, and ovary cancer types (Fig. 3B, Additional file 1: Table S5). The number and VAFs of SV events varied across samples while similar distributions were observed across different cancer types (Additional file 2: Figure S1). Most of the VAFs were distributed in 0 to 0.2 in each type of cancer. Next, according to SV discrimination results reported by expert review (see “Methods”), the random-forest decision model was successfully constructed using the training set and extracted SV features. The results showed that the random-forest decision model correctly predicted 344 true positive (TP−) and 559 true negative (TN−) SVs in the training set, 95 TP- and 130 TN-SVs in the testing set 1, respectively (Fig. 3C, Additional file 1: Table S4). An independent testing set involving 60 patients (Cohort 2, Fig. 3A, B) was further used and similar results were obtained (65 TP- and 106 TN-SVs were identified, Fig. 3C, Additional file 1: Table S4). The overall performance of the random-forest decision model indicated its high accuracy, high precision, high recall, and high F1 score of over 98.4% in the training and two testing sets (Fig. 3D).

### Validation of the SV detection framework based on standard materials and experiments

**Fig. 4:**
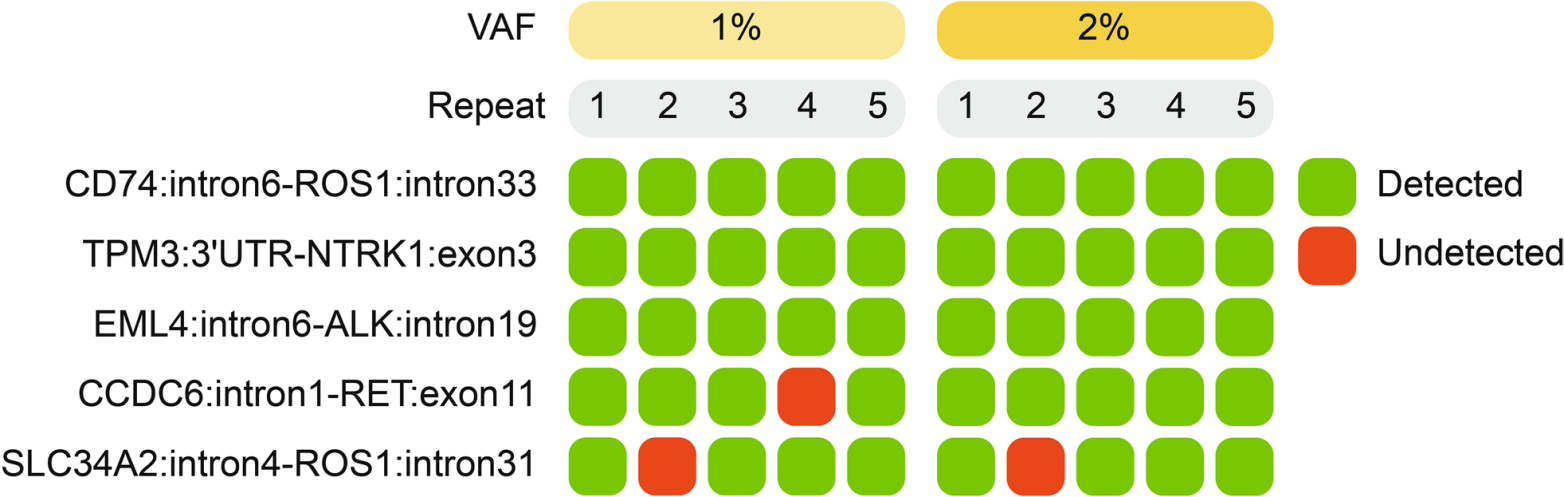
Performance validation of our framework in SV detection using standard materials

**Fig. 5:**
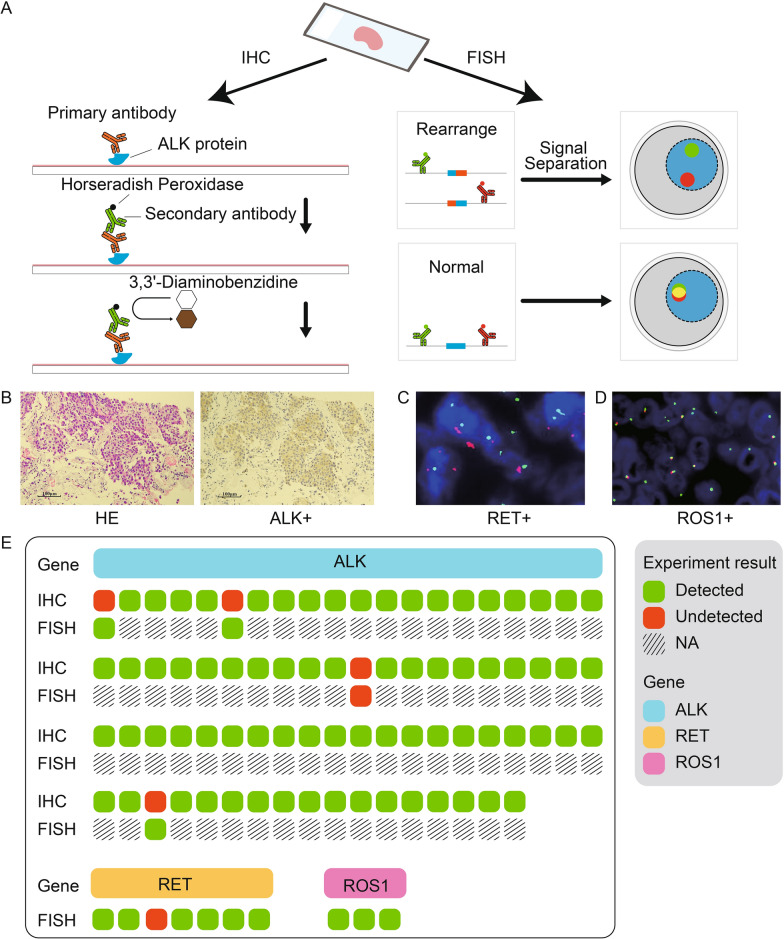
Performance validation of our framework using the IHC and FISH experiments. **A** Experimental flow chart of IHC (left) and FISH (right). **B** H&E staining of tumor areas (left) and positive staining with ALK fusion (right). **C** RET fusion positives by FISH. **D** ROS1 fusion positives by FISH. **E** Recall metric indicating the performance of our framework in SV detection by IHC and/or FISH experiments

In order to further verify the performance of the SV detection framework, the tests based on standard materials and IHC and/or FISH experiments were carried out. The current standard materials contained five custom SVs (Additional file 1: Table S2) with two VAF gradients (1% and 2%). Five biological replicates for each VAF gradient were tested independently. The results showed that the average detection rates were 92% and 96% of SVs when VAF gradients were equal to 1% and 2%, respectively (Fig. 4), demonstrating the robustness of the framework in standard materials. Next, 87 tumor samples (Cohort 3) from lung cancer patients were collected for targeted panel sequencing and IHC and/or FISH experiments (see “Methods”, Fig. 5A). As the most common fusion genes in lung cancer, *ALK*, *RET*, and *ROS1* were selected for analysis. Through the comparison with IHC and/or FISH experiment results, the detection rate of the framework was 98.70% (76/77), 85.71% (6/7), and 100% (3/3) for *ALK*, *RET*, and *ROS1* gene fusion, respectively (Fig. 5B–E), further suggested the accuracy of the SV detection framework.

## Discussion

Numerous studies have reported that the detection of SVs in cancer patients is crucial for diagnosis and targeted therapies. As noted in the literature, existing SV detection algorithms can provide relatively high-accuracy calls for SVs but their application in clinical practice is limited. In this study, we applied the tailored ‘two-step’ strategy for clinically-relevant SV detection that combines an optimal SV caller and a random-forest-based decision model and significantly improved the accuracy of SV detection in clinically targeted NGS panel data. The robustness of the framework was verified by using standard materials and IHC and/or FISH experiments.

Generally, determining whether SV is a true or false positive depends on various factors, such as the characteristics of soft-clip/split reads, the number of reads, and signal strengths of the breakpoints. When constructing the features required by the random-forest decision model, we extracted information about features caused by the SV from multiple facets. These features can be divided into three categories, including (1) the fundamental character of reads within a 20 bp range upstream and downstream of the breakpoints, including min, max, etc.; (2) the information of breakpoints within simple repeat regions; (3) the quantity of split-reads and paired-reads, including high-quality variant pairs (DV) and high-quality variant junction reads (RV). Based on all extracted features, we successfully constructed a random-forest decision model. In addition, we used internal and external variation (an independent) datasets, to further verify the performance of the model. The results show that the random-forest decision model achieves quite excellent prediction effects on two variation datasets.

This study still has limitations. On the one hand, as numerous cancer diagnosis and treatment relevance SV detection projects are being carried out, an increasing number of clinical SVs will be updated in the CIViC database, potentially causing fluctuations in the genes contained in the current panel. On the other hand, although the current framework has demonstrated high accuracy across multiple levels of evaluation and validation, only a limited number of clinical samples were employed to evaluate the performance of SV detection. In this study, we used three main ways to verify the accuracy of SV results: the expert-reviewed results, the use of standard materials, and the verification by IHC and/or FISH experiments, all of which were often missing in the public database. So, limited by the lack of the SV benchmark data, genome data from the public database is currently not available for our model validation. Nevertheless, the use of more types of datasets to verify the accuracy of our framework is needed in the future.

## Conclusions

This study not only provides a valuable resource for clinically-relevant SV re-analysis but also offers an actionable guide to improve cancer-specific SV detection accuracy and clinical applicability. Using targeted sequencing affords a cost-efficient alternative for cancer patients, particularly those in economically disadvantaged households. In addition, we hope that our work will provide more insights for researchers into the development of more SV detection accuracy methods in other gene panel data.

### Supplementary Information

**Additional file 1: Table S1-S5.** **Table S1.** Custom SVs in variant simulation data. **Table S2.** Custom SVs in standards materials. **Table S3.** Evidence level of 27 selected genes by CIViC database. **Table S4.** The annotated and predicted information of SV in Cohort 1 and Cohort 2. **Table S5.** Cancer types of each patient in Cohort 1 and Cohort 2.

**Additional file 2: Figure S1.** The distribution of the number and VAF of SVs. (A) The distribution of the number of SVs in Cohort 1. (B) The distribution of the number of SVs in Cohort 2. (C) The distribution of the VAF of SVs in Cohort 1. (D) The distribution of the VAF of SVs in Cohort 2.

## Data Availability

All data generated or analyzed during this study are included in this published article [and its supplementary information files]. The raw data used in this study can be obtained by request to the corresponding author.

## Abbreviations

SV: Structural variant
IGV: Integrative Genomics Viewer
FDA: Food and Drug Administration
WGS: Whole-genome sequencing
WES: Whole-exome sequencing
IHC: Immunohistochemistry
FISH: Fluorescence in situ hybridization
VAF: Variant allele frequency
