# Supplementary material for: A benchmarking framework for the accurate and cost-effective detection of clinically-relevant structural variants for cancer target identification and diagnosis
Source: J Transl Med. 2024 Jan 16;22:65. doi: 10.1186/s12967-024-04865-w (PMC10792779; doi:10.1186/s12967-024-04865-w)
Supplement: Supplementary file 2 — Additional file 2: Figure S1. The distribution of the number and VAF of SVs. (A) The distribution of the number of SVs in Cohort 1. (B) The distribution of the number of SVs in Cohort 2. (C) The distribution of the VAF of SVs in Cohort 1. (D) The distribution of the VAF of SVs in Cohort 2. [file 12967_2024_4865_MOESM2_ESM.docx]

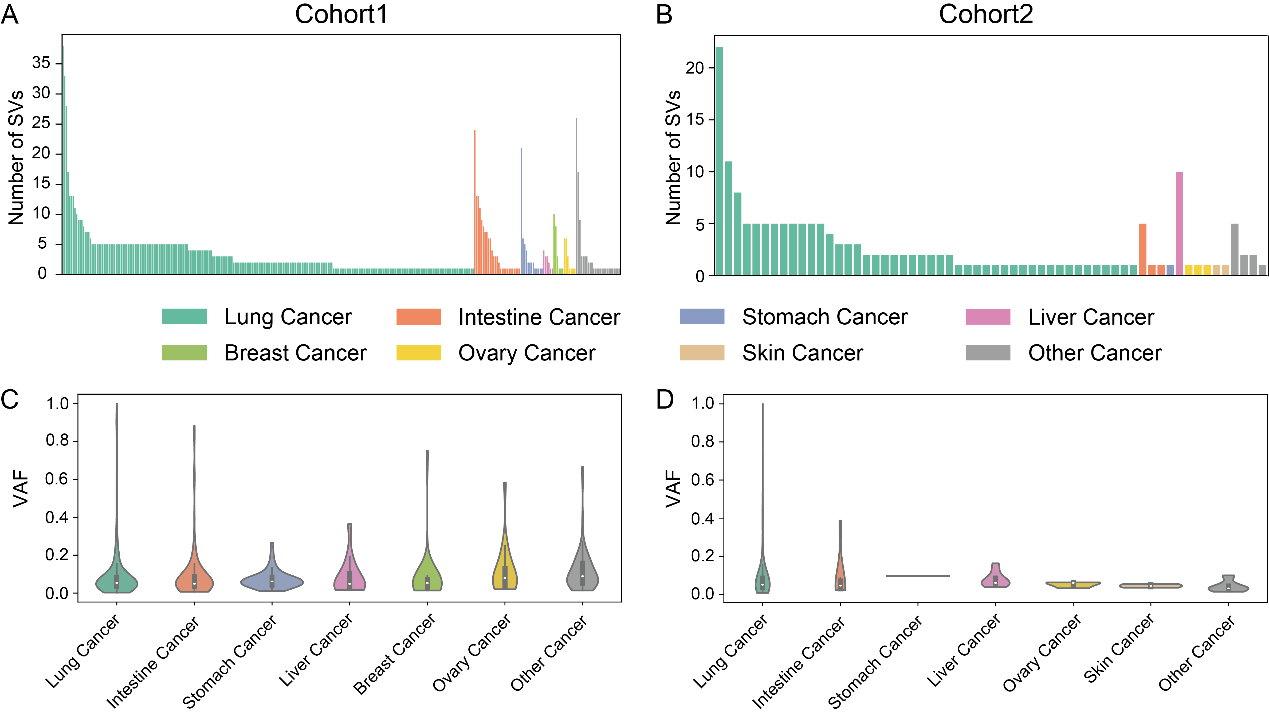


**Figure S1.** The distribution of the number and VAF of SVs. (A) The distribution of the number of SVs in Cohort 1. (B) The distribution of the number of SVs in Cohort 2. (C) The distribution of the VAF of SVs in Cohort 1. (D) The distribution of the VAF of SVs in Cohort 2
